# Analytical and Computational Methods for the Estimation of Drug-Polymer Solubility and Miscibility in Solid Dispersions Development

**DOI:** 10.3390/pharmaceutics11080372

**Published:** 2019-08-01

**Authors:** Djordje Medarević, Jelena Djuriš, Panagiotis Barmpalexis, Kyriakos Kachrimanis, Svetlana Ibrić

**Affiliations:** 1 Department of Pharmaceutical Technology and Cosmetology, Faculty of Pharmacy, University of Belgrade, Vojvode Stepe 450, 11221 Belgrade, Serbia; 2 Department of Pharmaceutical Technology, Faculty of Pharmacy, Aristotle University of Thessaloniki, 54124 Thessaloniki, Greece

**Keywords:** solid dispersions, miscibility, solubility, thermodynamic modeling, phase diagram, molecular dynamics simulation, thermal analysis, spectroscopic techniques

## Abstract

The development of stable solid dispersion formulations that maintain desired improvement of drug dissolution rate during the entire shelf life requires the analysis of drug-polymer solubility and miscibility. Only if the drug concentration is below the solubility limit in the polymer, the physical stability of solid dispersions is guaranteed without risk for drug (re)crystallization. If the drug concentration is above the solubility, but below the miscibility limit, the system is stabilized through intimate drug-polymer mixing, with additional kinetic stabilization if stored sufficiently below the mixture glass transition temperature. Therefore, it is of particular importance to assess the drug-polymer solubility and miscibility, to select suitable formulation (a type of polymer and drug loading), manufacturing process, and storage conditions, with the aim to ensure physical stability during the product shelf life. Drug-polymer solubility and miscibility can be assessed using analytical methods, which can detect whether the system is single-phase or not. Thermodynamic modeling enables a mechanistic understanding of drug-polymer solubility and miscibility and identification of formulation compositions with the expected formation of the stable single-phase system. Advance molecular modeling and simulation techniques enable getting insight into interactions between the drug and polymer at the molecular level, which determine whether the single-phase system formation will occur or not.

## 1. Introduction

Rise in the number of poorly soluble drugs is accompanied by simultaneous progress in the development of techniques for improving solubility and bioavailability of these drugs, which include but are not limited to: formation of salts and soluble prodrugs, particle size reduction up to nano-size range, using of cosolvents or surfactants in the formulation, complexation with cyclodextrins, formulation of micro or nanoemulsions, and solid dispersions. Solid dispersions, as systems where the drug is dispersed within the polymeric matrix in the crystalline or amorphous state, or dissolved in the polymeric matrix, have been proved to be one of the most successful approaches for overcoming drugs’ poor solubility and bioavailability [1,2,3,4,5,6]. Even though since the introduction of solid dispersions in 1961, by Sekiguchi and Obi [7], thousands of studies have proved their benefits, both in vitro and in vivo, only a few such formulations have appeared on the market up to date. One of the main reasons for this is certainly the difficulty of ensuring long-term product stability due to phase separation between the drug and the polymer and/or drug recrystallization from the initial amorphous form that can cause unacceptable variations in drug dissolution rate and oral bioavailability. It has been well established that apart from differentiation whether the drug is present in the crystalline or amorphous form within the polymeric matrix, it should be determined whether the drug forms single-phase system with amorphous polymer or system separates into drug-rich and polymer-rich phases. It has been shown that the maximum improvement of drug dissolution rate and maintenance of long-term formulation stability are achieved if the formation of a single-phase system occurs [8,9]. Otherwise, if the phase separation occurs, properties of pure components will dominate in the respective phases, and polymer effect on inhibition of drug molecular mobility and reduction of driving force for crystallization will be diminished. Additionally, separation into drug-rich and polymer-rich phases will lead to the fast dissolution of the polymer phase leaving undissolved drug phase [10,11]. Currently available analytical techniques can distinguish between drug and polymer domains of different size, but only at the moment of analysis, which does not guarantee that initially single-phase system will maintain this structure during the whole storage period. Also, direct measurement of drug-polymer miscibility or solubility of the drug in the polymer is challenging due to the high viscosity of polymers below glass transition temperature (*T_g_*), which makes it difficult to achieve equilibrium in the drug-polymer system in the glassy state [12,13]. Only in the last 10 years, it has been recognized that the evaluation of thermodynamics of drug-polymer mixing should be included in the rational design of solid dispersion formulations. Thermodynamic models, initially developed for polymer-polymer and polymer-solvent systems, have been successfully adapted to drug-polymer systems and showed the good prediction of drug-polymer miscibility and solubility of the drug in the polymeric matrix [8,11,14,15,16,17,18,19,20]. Although the terms solubility and miscibility are sometimes used with confusion, they can be distinguished, since the term solubility describes the ability of a polymer to dissolve a crystalline drug, while miscibility describes the ability of an amorphous drug to mix with an amorphous polymer giving a single-phase system [11,12]. Although only below the drug solubility limit in the polymer, solid dispersion systems are stable without any concern for drug crystallization, the low solubility of most drugs in the common polymers limits formulation of solid solutions only to very low dose drugs. Therefore, particular efforts are invested to estimate the miscibility of the drug with the polymer, which is always greater than the drug solubility in particular polymer, and below miscibility limit, only large temperature and/or composition fluctuations can destabilize system toward drug crystallization. Thermodynamic modeling allows estimation of the free energy of mixing between the drug and the polymer, with regards to formulation composition and temperature, i.e., whether mixing between the drug and polymer is spontaneous or not at a particular drug:polymer ratio and temperature. This approach, based on well-known Florry-Huggins theory [21], allows construction of temperature-composition phase diagrams, which separates stable, metastable, and unstable regions and helps formulation scientists to choose appropriate formulation compositions and processing conditions during solid dispersions preparation. Apart from the estimation of formulation stability, usage of thermodynamic modeling is particularly beneficial in the early stages of formulation development, when a limited amount of material is available, since totally immiscible formulations can be rejected at this stage, saving both materials and time. In this review, we have given an overview of the currently available methodology for the estimation of miscibility between the drug and polymer as well as solubility of the drug in the polymer. Although in the text, methods are separated into analytical methods and computational methods (based on thermodynamic modeling and molecular modeling and simulations), one should be aware that estimation of the drug-polymer miscibility and the solubility of the drug in the polymer is a complex problem, requiring multi-methodological approach.

## 2. Analytical Techniques for the Assessment of Drug-Polymer Solubility/Miscibility

Once a solid dispersion system is formed, the solid-state characterization is performed using various techniques to estimate the physical state of the drug and/or potential interactions, which can be attributed to the miscibility, with the selected polymers. In the case of the well-mixed system, only one phase exists since the system components (i.e., drug and the polymer) are intimately mixed at the molecular level. On the other hand, the presence of at least two different phases shows that components are immiscible. These differences are reflected in the physical properties and can be analyzed using a variety of analytical techniques for solid-state characterization.

### 2.1. Thermal Techniques

Thermal characterization, using Differential Scanning Calorimetry (DSC) or Modulated-temperature Differential Scanning Calorimetry (M-DSC), is used to determine the solid-state of the drug and possible drug-polymer interactions in the prepared solid dispersions. M-DSC enables determination of both the specific heat capacity and the heat flow data from a kinetically controlled process [22]. Polymers used for the preparation of the solid dispersions are usually amorphous and thermoplastic with specific glass transition temperatures (*T_g_)*. If the selected drug is crystalline, it usually preserves this state in physical mixtures with the polymer, which is evident on the DSC thermogram of the physical mixture as sharp endothermic peak(s), corresponding to the drug melting point. However, the absence of drug-specific endothermic melting peak(s) may suggest either that the drug is present in the solid dispersion in its amorphous state (drug forms single-phase or multi-phase system with polymer), or it is solubilized during DSC analysis by the polymer (or other excipients) used for the preparation of solid dispersions. Furthermore, shifts in the polymer *T_g_* may also occur, which is also indicative of molecular interactions between the drug and the polymer [23]. Since solid dispersions, with miscible drug and polymer, create a single-phase amorphous system, single *T_g_* peak is considered as the marker of the miscible drug-polymer system [24]. When the two components are miscible, the single *T_g_* of the formed solid dispersion lies between the *T_g_*s of the individual components [25].

Melting point depression is one of the most common analytical methods for the assessment of the drug and polymer miscibility. The changes in the onset of the melting endotherm or the drug melting enthalpy are monitored as a function of the polymer amount in the prepared solid dispersions. In the case of the miscible system, a decrease in the drug melting point(s) and/or enthalpies is expected with the increase of the polymer amount. If the DSC scan represents separate glass transition points, *T_g_*, specific of the drug and the polymer, it is an indication that the prepared solid dispersions do not constitute a miscible system, i.e., two individual phases are present [23]. Therefore, immiscibility is usually manifested as the phase separation, i.e., the existence of crystalline or amorphous domains within the polymer or two separated amorphous domains. There may also be a gradient of drug concentrations in different regions of the dispersion [26].

Several different approaches have been established to estimate *T_g_* of drug-polymer mixtures based on the known mixture composition. Certainly, the most widely used equation for the estimation of mixture *T_g_* (*T_g_^mix^*) is the Gordon-Taylor equation [27]:

(1) $$T_{g}^{mix}=\frac{w_{1}T_{g1}+K\;w_{2}T_{g2}}{w_{1}+K\;w_{2}}$$

where *w* and *T_g_* are weight fraction and glass transition temperature of each component, respectively, while subscripts 1 and 2 represent components with the lowest and the highest *T_g_*, respectively. Constant *K* is originally defined as a parameter whose value depends on the change of thermal expansion coefficient of the components upon their transformation from glassy to the rubbery state during glass transition. This constant is usually calculated using Simha-Boyer rule [28]:

(2) $$K=\frac{\rho_{1}T_{g1}}{\rho_{2}T_{g2}}$$

where *ρ_1_* and *T_g1_* are the density and the glass transition temperature of the amorphous component with the lowest *T_g_*, respectively, and *ρ_2_* and T_g2_ are the density and glass transition temperature of the amorphous component with the highest *T_g_*, respectively. Couchman and Karasz [29] proposed a thermodynamic model to predict the *T_g_* of mixtures:

(3) $$ln\;T_{g}{}^{mix}=\frac{w_{1}\Delta C_{p1}ln\;T_{g1}+w_{2}\Delta C_{p2}ln\;T_{g2}}{w_{1}\Delta C_{p1}+w_{2}\Delta C_{p2}}$$

where Δ*C_p_* is a change in heat capacity of the component between liquid-like and glassy state. Another approach for the prediction of mixture *T_g_* represents Fox equation [30]:

(4) $$\frac{1}{T_{g}^{mix}}=\frac{w_{1}}{T_{g1}}+\frac{w_{2}}{T_{g2}}$$

When using these theoretical approaches to predict mixture *T_g_*, one should be aware of some inherent limitations of these methods. These approaches assume the absence of specific interactions between components (i.e., ideal mixing behavior is assumed), ideal volume additivity of the components at *T_g_*, and linear change in volume with temperature [31,32]. Therefore, the presence of interactions between components will result in deviations between experimentally observed *T_g_* of the mixture and those predicted by previously described models. Negative deviation of experimental *T_g_* from predicted one can indicate that cohesive interactions between individual components are more pronounced than adhesive drug-polymer interactions, as observed for indomethacin-polyvinylpyrrolidone (PVP) system [33,34]. Positive deviation of experimental *T_g_* from predicted one indicates that drug-polymer interactions are stronger than drug-drug and polymer-polymer interactions. This effect has been observed for numerous solid dispersion systems, such as indomethacin-Eudragit^®^ E [34,35], lapatinib-hydroxypropylmethylcellulose phthalate (HPMCP) [36], nimodipine-PVP [37]. However, the presence of positive or negative deviations of experimental from predicted *T_g_* is not a reliable indicator whether adhesive or cohesive interactions are predominant. This is demonstrated for curcumin-hydroxypropylmethylcellulose (HPMC) solid dispersions, where the negative deviation of experimental *T_g_* from predicted one is observed, even though the presence of drug-polymer intermolecular interactions is proved by FT-IR spectroscopy [38]. An additional limitation of the presented models is that they do not take into account entropic contribution to the drug-polymer mixing. It should be also noted that the chosen experimental conditions can influence the measured values of *T_g_*. Gordon-Taylor equation has been adapted for ternary solid dispersions (Equation (5)); however, above-mentioned basic assumptions of this model significantly limit its application for ternary systems, making difficult to draw any conclusions from the obtained results:

(5) $$T_{g}^{mix}=\frac{w_{1}T_{g1}+K_{1}w_{2}T_{g2}+K_{2}w_{3}T_{g3}}{w_{1}+K_{1}w_{2}+K_{2}w_{3}}$$

(6) $$K_{1}=\frac{\rho_{1}T_{g1}}{\rho_{2}T_{g2}}$$

(7) $$K_{2}=\frac{\rho_{1}T_{g1}}{\rho_{3}T_{g3}}$$

**Figure 1:**
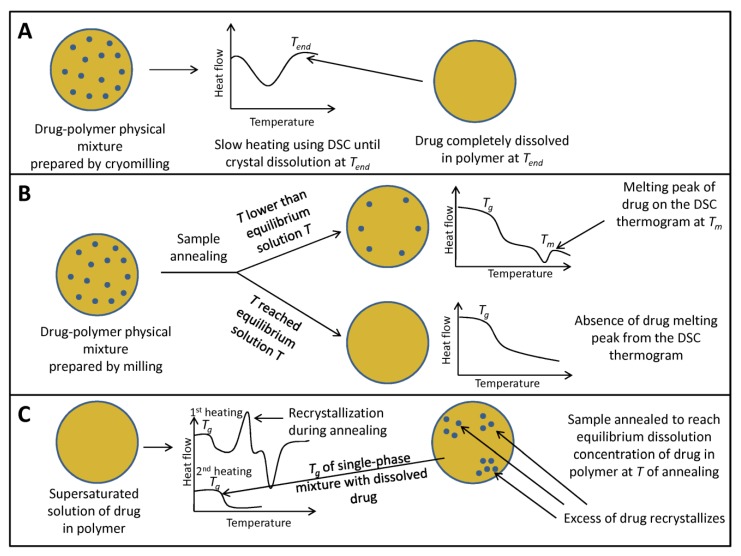
Schematic drawing of methods for the determination of drug solubility in polymer described by (**A**) Tao et al. [13], (**B**) Sun et al. [42], and (**C**) Mahieu et al. [43] (*T*—temperature, *T_g_*—glass transition temperature, *T_end_*—end temperature of drug dissolution, *T_m_*—melting temperature).

It has been reported that some microstructural phase separations could not be detected by the DSC method due to its resolution limitation (~30 nm) [39]. If the drug and the polymer have similar *T_g_* values, then it is also difficult to estimate their miscibility using DSC studies [26]. Another limitation of the conventional DSC analysis is the fact that the drug may dissolve in the molten polymeric material below its melting point, which may be mistakenly considered as solubility/miscibility [40]. If the crystalline drug dissolves in the molten polymer during heating, it is better to use fast DSC analysis (such as M-DSC) because higher heating rates may hinder the drug dissolution process [26]. Fule and Amin [41] used M-DSC studies to investigate whether the absence of drug melting endotherm in the DSC scan is a consequence of the presence of drug amorphous form, or drug dissolution within the molten excipients during DSC scan. They demonstrate that the endothermic peak, corresponding to the melting of crystalline drug, broadens during the first heating cycle and disappears in the second heating cycle of M-DSC analysis. On the other hand, melting peak of the drug is absent on the DSC thermogram since the crystalline drug gradually dissolves in the molten polymers during conventional DSC heating process, giving false evidence of the presence of amorphous drug [41]. Tao et al. [13] have used slow heating rates for DSC measurements and extrapolated the temperature of the final dissolution of the crystalline drug to zero heating rate to determine the solubility of the small molecule crystalline drugs in the polymer. If mixture containing known composition of the crystalline drug (*x*) is heated, the broad endothermic peak occurs due to the drug dissolution, and the drug solubility in the polymer is defined as *x* at the end temperature of drug dissolution (*T_end_*). Specifically, cryogenic milling is used for sample preparation to ensure uniform mixing and facilitate determination of dissolution endpoint. However, even at a low heating rate (0.1 °C/min), the available time during DSC analysis may not be sufficient to reach equilibrium (i.e., *T_end_* is higher than equilibrium solution temperature), and solubility of the drug in the polymeric matrix may be underestimated. This problem is particularly pronounced at temperatures close to *T_g_*, due to high polymer viscosity, which causes the time for reaching equilibrium to be much higher, compared to the timescale of the DSC scan. Therefore, this method was further refined by Sun et al. [42] who proposed a method where the drug-polymer mixture is annealed during 4–10 h near an equilibrium solution temperature followed by the scan at standard scanning speed (10 °C/min) to detect the presence of undissolved drug crystals. If the annealing temperature is lower than the equilibrium solution temperature, the melting endotherm will appear in the heating scan due to the presence of undissolved crystals. By annealing at different temperatures, boundaries of equilibrium solution temperature can be determined. Although this method enables determination of solubility at a temperature closer to *T_g_* and improves sensitivity to detect residual drug crystals, it is still considerably time-consuming and requires several experiments for only one point in the solubility plot. The method proposed by Mahieu et al. [43] is based on the generation of the supersaturated solid solution of drug in polymer and further induction of demixing by annealing above *T_g_*. The equilibrium concentration of a dissolved drug is subsequently determined by measuring *T_g_* of the annealed mixture and further calculation from Gordon-Taylor equation (Equation (1)), which gives the relationship between *T_g_* of the mixture and mixture composition. This method is much faster, since the demixing process is faster than dissolution, due to enhanced mobility in the supersaturated system caused by plasticization effect of drug molecules, and only one experiment is required to generate one point in the drug-polymer solubility plot. Schematic drawing of methods for the determination of drug solubility in the polymer, described in the references [13], [42], and [43] is given in Figure 1. Tian et al. [44] recently proposed an improved method for the determination of equilibrium drug solubility within the polymeric matrix and the determination of the solid-liquid transition curve. In this method, a mixture of drug and polymer is firstly undergone to hot-melt extrusion and then subject to isothermal annealing at elevated temperatures (above *T_g_* of polymer and below the melting temperature (*T_m_*) of the drug) during 24 h. High-speed DSC (Hyper DSC) analysis with a heating rate of 200 °C/min is used to detect the presence of undissolved drug crystals remained after sample annealing. This method should provide a more reliable determination of drug solubility within the polymer as long annealing process provides sufficient time for dissolution of drug crystals and overcoming of high polymer viscosity, which can delay completion of drug dissolution. High heating rate after sample annealing provides greater sensitivity to detect melting endotherm of remaining drug crystals, compared to usual DSC heating rates (1–10 °C/min), with the lower possibility that drug crystal will dissolve during DSC scan, leading to overestimation of drug equilibrium solubility in the polymer [44].

Thermally stimulated depolarization current (TSDC) is a method for measurement of dielectric properties through thermally stimulated depolarization of materials molecules. This technique can also be used to study the miscibility of the drug and the selected polymer [45]. Shmeis et al. [46] have compared TSDC and DSC for the assessment of the miscibility of the novel drug substance and PVP and demonstrated the superiority of the TSDC method. At higher drug loads, the saturation level of the drug within the polymer has been only possible to be detected by the TSDC method of analysis.

### 2.2. Spectroscopic Techniques

Apart from the thermal methods, miscibility within the solid dispersions is often analyzed by spectroscopic techniques. Fourier Transform Infrared (FT-IR) Spectroscopy can be used to study specific interactions between the polymer and the drug functional groups. Hydrogen bonding is a predominant mechanism for the stabilization of the miscible drug-polymer systems. When such bonds are formed, subtle changes in the FT-IR spectra are visible [23]. IR spectroscopy with principal component analysis can be utilized to verify drug-polymer mixing at the molecular level [47].

Taylor and Zografi [33] have criticized the traditional approach where the spectra of the pure crystalline drug and solid dispersion are compared to assess changes in the crystallinity of the drug and potential for miscibility with the polymer. More detailed studies on the amorphous structures should be performed, using the spectra of the amorphous form of the drug as the reference [33].

Solid-State Nuclear Magnetic Resonance (SSNMR) spectroscopy is often employed for structural analysis and the assessment of interactions between the molecules. ^1^H-NMR spin-lattice relaxation measurements can be used for the assessment of the drug-polymer miscibility [48]. The main benefit of this technique is that it can detect phase-separated domains with size below the detection limit of DSC. The miscibility of the drug-polymer and size of the phase-separated domains are estimated based on the spin-lattice relaxation times in the laboratory (^1^H T_1_) and rotating frames (^1^H T_1ρ_). In the phase-separated system, protons in each phase will have their relaxation times, so individual relaxation times of drug and polymer will be observed. On the other hand, in a single-phase system, spin diffusion should average individual relaxations, resulting in uniform average relaxation time, different from relaxation time measured for pure components [36,49,50]. Single ^1^H T_1_ in the drug-polymer system indicates miscibility down to 20–50 nm domain size, while single ^1^H T_1ρ_ indicates miscibility with domain size below 5 nm [51]. Formation of single-phase solid dispersions with indomethacin and Eudragit^®^ E, with drug loading up to ~50%, has been shown based on similar ^1^H T_1_ and ^1^H T_1ρ_ values for the drug and polymer [50]. Miscibility of the selected drug (nifedipine) and polymers (PVP and HPMC) is appointed by mono-exponential spin-lattice relaxation decay for measurements of solid dispersions in the rotating frame [48]. Geppi et al. [52] have used several high-resolution solid-state NMR techniques to confirm the miscibility of ibuprofen and Eudragit^®^ L100 and the chemical nature of their interactions. Phase separation, which is indicative of the immiscible system, has been also assessed by NMR relaxometry study [26].

X-ray Photon Spectroscopy (XPS) is an advanced surface analysis technique, which can also be used to assess the magnitude of the intermolecular interactions between the drug and polymer, which are indicative of the miscibility within the system [24]. Due to the drug-polymer interactions in the obtained spectra, new peaks are formed, and several bond peaks are shifted. It has been demonstrated that drug-polymer interactions observed through XPS analysis are directly related to their miscibility [24].

### 2.3. X-ray Powder Diffraction (XRPD)

X-ray Powder Diffraction (XRPD) technique, in general, can be used to assess the crystalline state of the material. It is to be expected that solid dispersions prepared from miscible drug-polymer systems are amorphous and lack typical crystalline patterns in an X-ray diffractogram. Newman et al. [39] have developed the XRPD method coupled with the computation of pair distribution functions (PDF) to analyze miscibility in the drug-polymer systems. A lack of agreement of the PDF profiles of the solid dispersions and individual components indicates that the mixture with a unique PDF is miscible [39]. The method proposed by Newman et al. [39] has revealed the superiority of XRPD studies over the DSC analysis for the assessment of the drug-polymer miscibility due to an inability of the DSC technique to detect *T_g_* values for amorphous domains smaller than 30 nm. XRPD with PDF and pure curve resolution method (PCRM) analysis may be used to verify drug-polymer mixing for both completely and partially miscible systems. These techniques are especially useful for the examination of miscibility when DSC measurements are inconclusive or yield variable results [47].

### 2.4. Microscopic and Imaging Techniques

Methods for the visual analysis of solid dispersion samples are also of great importance to study the solubility/miscibility within the solid dispersion systems. Scanning electron microscopy (SEM) or transmission electron microscopy (TEM) studies of prepared solid dispersions may be indicative of intrinsic miscibility of the drug and polymer. The surface and cross-section morphological features of prepared solid dispersions are studied to analyze whether the miscible system is formed, usually in addition to DSC and XRPD analyses. An example of the assessment of the drug and polymer miscibility using SEM demonstrates that solid dispersions appear to be agglomerated with a rough surface, which is attributed to the miscibility of the drug and polymer [41].

3D surface Atomic Force Microscopy (AFM) imaging analysis of solid dispersions is used to further elucidate drug-polymer surface morphology and interactions [41]. Although AFM provides nanoscale resolution, which is desirable in miscibility evaluation, it cannot provide information regarding chemical composition in different regions of analyzed samples. To overcome this drawback, AFM imaging is coupled with the source of IR radiation (nanoscale infrared spectroscopy—nano IR, AFM-IR) or heating source (nanoscale thermal analysis—nanoTA). In the AFM-IR technique, light from IR source is focused on the contact area between the sample and AFM tip. Absorption of IR radiation causes thermal expansion of the sample, which induces cantilever oscillation. The spectrum of the amplitude of cantilever oscillations as a function of IR wavelength is unique for each sample and provides information about chemical composition in the analyzed sample. In the nanoscale thermal analysis, AFM probe is heated and moved along the region of interest and, when the thermal event occurs, a region of the sample softens and AFM probe penetrates the sample. By measuring thermal properties across the sample surface, nanoscale thermal analysis enables evaluation of whether a system is single-phased or phase separation occurred. Nanoscale infrared spectroscopy and nanoscale thermal analysis have been successfully used to evaluate miscibility between telaprevir and three different polymers [53]. Crystalline structures may also be visually observed by polarized light microscopy (PLM) due to the characteristic appearance of birefringence of crystalline structures, which are formed in immiscible systems [54]. Miscibility within the drug-polymer system can be further confirmed by the hot stage microscopy (HSM) [55], which is often coupled with polarized light microscopy.

Raman mapping (or Raman spectral imaging) is a method whereby detailed chemical images are generated from samples’ Raman spectra. Raman mapping may also be used to investigate the drug crystallinity [56]. Analysis of small-size samples through micro-Raman mapping can be used to detect the phase separation in systems in which multiple glass transition events cannot be resolved by DSC [57]. The main benefit of using Raman mapping in the evaluation of drug-polymer miscibility is providing information regarding the chemical composition of phase-separated domains. Qian et al. [58] have demonstrated the superiority of a Raman mapping method over conventional DSC and XRPD studies for analysis of the drug-polymer miscibility within the two batches of amorphous solid dispersion systems that exhibit different physical stability against crystallization over time. They demonstrated that single distinctive *T_g_* might not always be a reliable indicator of homogeneity and optimal stability since Raman maps of the less stable systems are indicative of wide distribution ranges of the drug concentration [58].

Thermal analysis by structural characterization (TASC) is a novel thermal analysis technique that combines image analysis with hot stage microscopy. TASC technique is based on the algorithm that converts any change in the sample appearance during heating into a quantified signal, i.e., TASC curve [59]. Suitability of TASC as a technique for fast screening of the drug-polymer miscibility by evaluating the melting point depression has been demonstrated for felodipine and ten commonly used polymers in solid dispersion formulations [60]. Fast analysis, high sensitivity, and the requirement of a small amount of sample make this technique very attractive in the early stage of solid dispersion formulation development; however, further studies are necessary to confirm the usefulness of this technique.

Martinez-Marcos et al. [55] have highlighted the potential of the novel technique, micro-computed tomography (μ-CT), to be used for the characterization of internal materials properties. This technique enables X-ray imaging in three dimensions. X-ray micro-computed tomography and TASC technique are used in conjunction with conventional thermal, microscopic, and spectroscopic techniques to analyze the miscibility of felodipine with several excipients [61].

### 2.5. Other Techniques

Crowley and Zografi [62] have estimated the miscibility of PVP and three hydrophobic drugs through water vapor absorption studies. They demonstrated that interactions in amorphous dispersions affect the water uptake properties of the individual components [62]. Liu et al. [35] have reported on the potential for application of rheological measurements to accompany thermal and spectroscopic analysis for the assessment of the drug and polymer miscibility. Gupta et al. [63] have proposed that if the viscosity versus temperature plots for different drug concentrations are parallel to each other (without observable drug melting transition), it is indicative of complete drug-polymer miscibility.

### 2.6. Techniques Used in Combination

It might be of great interest to use several analytical techniques for investigation of the miscibility of the selected drug-polymer system. Rumondor et al. [47] have demonstrated that DSC, FT-IR spectroscopy, and XRPD analysis provide complementary results to each other. Marsac et al. [64] have used DSC, AFM, and TEM techniques to study the effect of temperature and moisture on the miscibility within the drug-polymer solid dispersions. It is also of great interest to study ternary systems, i.e., to estimate the solubility/miscibility of the drug within the polymer mixtures. Janssens et al. [65] have analyzed the miscibility in ternary systems of itraconazole with polyethylene glycol (PEG) and HPMC polymer blends, of different molecular weights, using M-DSC and XRPD. Miscibility in ternary polymer-drug-surfactant systems hydroxypropylmethylcellulose acetate succinate (HPMCAS)-itraconazole-Soluplus^®^ has been also analyzed using DSC, XRPD, and PLM [66]. Parikh et al. [67] have proposed the preparation of the film-casted samples to investigate miscibility of the drug and the polymer using techniques, such as DSC, XRPD, and PLM. Several analytical techniques are also used to investigate miscibility of felodipine with polymer blends used for the fused deposition modeling 3D printing [68]. Examples of usage of different analytical methods for the estimation of drug-polymer solubility/miscibility are given in Table 1.

## 3. Computational Methods for the Assessment of Drug-Polymer Solubility/Miscibility

### 3.1. Solubility Parameters

The early phase of formulation development requires fast screening methods capable of making rough differentiation between the drug-polymer immiscible systems and the drug-polymer systems that are likely to be miscible. The usage of solubility parameters, as a purely theoretical-based approach, perfectly fits this description since it enables evaluation of the drug-polymer miscibility based on only drug’s and polymer’s chemical structures and without the need for performing even single experiment. The concept of solubility parameters has been introduced by Hildebrand [76,77] and Scatchard [78] who have linked solubility of the solute in different solvents with cohesion energy density.

Cohesion energy is defined as the increase in internal energy per mole of a substance if all of the intermolecular forces are eliminated, i.e., cohesion energy represents the strength of attractive forces of constituent molecules in the substance. For low molecular weight molecules, cohesion energy (*E_coh_*) can be calculated from experimentally determined heat of vaporization using the following equation [79]:

(8) $$E_{coh}=\Delta H_{vap}-p\Delta V\approx \Delta H_{vap}-RT$$

where Δ*H_vap_* is molar heat of evaporation, *p* is pressure, Δ*V* is the volume change, *R* is the universal gas constant, and *T* is temperature.

In the concept proposed by Hildebrand, solubility parameter (*δ*) is calculated as a square root of cohesive energy density:

(9) $$\delta =\sqrt{\frac{E_{coh}}{V}}$$

Since it is not possible to determine the heat of vaporization for polymers, *δ* for these high molecular weight molecules can be determined by indirect methods, such as dissolving or swelling of polymers in series of solvents of known *δ* [80,81], measurements of polymers viscosity in the solvents of known *δ* [82], or using inverse gas chromatography [83]. Because these methods are time and material consuming, several group contribution methods are developed, which enable calculation of solubility parameters from the knowledge of the molecule’s chemical structure. The basic postulate of group contribution methods is that properties of the polymer can be estimated by summation of the contributions of its structural fragments. Although there are several group contribution methods for the estimation of the cohesion energy of the polymers, in the following text we have given an overview of the few of them, which are the most commonly used. One of the earliest group contribution methods for the estimation of Hildebrand solubility parameters has been proposed by Small [84] who defined parameter *F = (E_coh_V)^1/2^*, called molecular attraction constant, and provided values of this parameter for numerous structural groups. According to Small’s group contribution method, the Hildebrand solubility parameter can be estimated by summation of *F* for structural fragments of the molecule, using the following equation:

(10) $$\delta =\frac{\sum F}{V}$$

This system has been further refined by Hoy [85] and Hoftyzer and Van Krevelen [86] who provided refined and updated tables for group contributions to the overall *F* value of the molecule. Fedors [87] proposed a slightly different concept that provides contributions of a much larger number of structural groups to both *E_coh_* and volume of the molecule. According to Fedors’ method, the Hildebrand solubility parameter can be estimated according to the equation:

(11) $$\delta ={\left(\frac{\sum_{i}\Delta e_{i}}{\sum_{i}\Delta v_{i}}\right)}^{1/2}$$

where Δ*e_i_* and Δ*v_i_* are the additive atomic and group contribution for the energy of vaporization and molar volume, respectively.

The specificity of this approach is that it offers the way for estimating not only *E_coh_* but also volume from group contributions and allows estimation of the temperature dependence of *δ* from the knowledge of density-temperature dependence.

Although the introduction of the Hildebrand solubility parameter resulted in huge progress in studying solute-solvent interactions, its application is limited to the systems with predominant dispersion force between molecules (non-polar molecules). Therefore, this concept was further extended by Hansen [88], who defined three-dimensional Hansen solubility parameter, which applies to substances that, in addition to dispersion forces, also interact by hydrogen bonding and polar forces. Total cohesion energy (*E_coh_*) is redefined as the sum of the contributions from dispersion forces (*E_d_*), polar forces (*E_p_*), and hydrogen bonding (*E_h_*):

(12) $$E_{coh}=E_{d}+E_{p}+E_{h}$$

Accordingly, the three-dimensional solubility parameter can be expressed as follows:

(13) $$\delta^{2}=\delta_{d}^{2}+\delta_{p}^{2}+\delta_{h}^{2}$$

where *δ_d_*, *δ_p_,* and *δ_h_* are dispersion, polar, and hydrogen bonding partial solubility parameters, respectively.

Several group contribution methods have been developed to estimate the Hansen solubility parameter. Two most widely used group contribution methods have been developed by Hoftyzer and Van Krevelen [86] and Hoy [89]. According to the method proposed by Hoftyzer and Van Krevelen [86], partial solubility parameters are estimated using the following equations, and the total solubility parameter is calculated according to the Equation (13).

(14) $$\delta_{d}=\frac{\sum F_{di}}{V}$$

(15) $$\delta_{p}=\frac{\sqrt{\sum F_{pi}^{2}}}{V}$$

(16) $$\delta_{h}=\sqrt{\frac{\sum E_{hi}}{V}}$$

where *F_di_* is molar attraction constant due to dispersion component, *F_pi_* is molar attraction constant due to the polar component, *E_hi_* is hydrogen bonding energy, and *V* is molar volume.

If the two identical polar groups are presented in the symmetrical positions, *δ_p_* is reduced by multiplying value obtained using Equation (15) with one of the following correction factors: 0.5 for one plane of symmetry, 0.25 for two planes of symmetry, or 0 for more than two planes of symmetry. Besides, for molecules with several planes of symmetry, *δ_h_* is 0. The method developed by Hoy [89,90] is more complex and requires the usage of four additive functions and several auxiliary equations to estimate values of partial solubility parameters and the total solubility parameter. Equations that are used for calculations in Hoy’s method are given in Table 2 [79]. Although presented group contribution methods up to now have been enriched and provide a huge collection of group contributions for solubility parameters estimation with reasonable accuracy, further research in this field pointed out some drawbacks of this concept and provided its further refinement. It has been recognized that presented group contribution methods are quite simplified and neglected how groups are connected as well as interactions between adjacent groups and electron delocalization. Therefore, these methods are unable to distinguish between different isomers and estimate the same *δ* values for those molecules [91]. To overcome these drawbacks, Stefanis and co-workers developed a new group contribution system for the estimation of solubility parameters, through the division of molecules into the first order and second order groups. The first order groups describe the basic structure of the molecule, similar to the previously described group contribution methods. Additional second-order groups consist of two or three adjacent first-order groups, and they are based on the conjugation theory [92,93,94]. Conjugation is considered as one of the important stabilization mechanisms, whereby compounds with a higher number of conjugates are considered as more energetically stable. This theory, known as ABC approach (ABC-contribution of Atoms and Bonds to the properties of Conjugates), considers chemical compounds not as single structures but as hybrids of different conjugates formed by a different arrangement of valence electrons. Therefore, this method can capture intramolecular interactions between adjacent atoms as well as atoms separated by several bonds [91]. Second-order groups are formed from the two or three adjacent first-order groups and ranked based on their contribution to the standard enthalpy of formation. Structures that exhibit the highest contribution to the enthalpy of formation are considered as second-order groups for further calculations [92,93]. The basic equation for the estimation of Hansen solubility parameters according to Stefanis and Panayiotou group contribution method is given as follows [94]:

(17) $$\delta =\sum_{i}N_{i}C_{i}+W\sum_{j}M_{j}D_{j}$$

where *C_i_* is the contribution of the first-order group of type *i* that appears *N_i_* times in the compound and *D_j_* is the contribution of the second-order group of type *j* that appears *M_j_* times in the compound. The constant *W* is equal to 0 for compounds without second-order groups and equal to one for compounds, which have second-order groups.

Partial solubility parameters can be estimated by the following equations [94]:

(18) $$\delta_{d}={\left(\sum_{i}N_{i}C_{i}+\sum_{j}M_{j}D_{j}+959.11\right)}^{0.4126}{\left[MPa\right]}^{1/2}$$

(19) $$\delta_{p}=\left(\sum_{i}N_{i}C_{i}+\sum_{j}M_{j}D_{j}+7.6134\right){\left[MPa\right]}^{1/2}$$

(20) $$\delta_{h}=\left(\sum_{i}N_{i}C_{i}+\sum_{j}M_{j}D_{j}+7.7003\right){\left[MPa\right]}^{1/2}$$

whereby different equations are used for the cases where values of *δ_p_* and *δ_h_* are lower than 3 MPa^1/2^:

(21) $$\delta_{p}=\left(\sum_{i}N_{i}C_{i}+\sum_{j}M_{j}D_{j}+2.6560\right){\left[MPa\right]}^{1/2}$$

(22) $$\delta_{h}=\left(\sum_{i}N_{i}C_{i}+\sum_{j}M_{j}D_{j}+1.3720\right){\left[MPa\right]}^{1/2}$$

Stefanis and Panayiotou further proposed subdivision of *δ_h_* into acidic and basic components *δ_a_* and *δ_b_*, respectively, and extended the three-parameter Hansen solubility parameter to the four-parameter solubility parameter. The donor and acceptor parameters of the hydrogen bonds have been obtained by evaluation of third moments of sigma profiles of charge density distribution on the surface of molecules. These profiles for a large number of compounds are available in the software databases (COSMObase or VT Sigma Profile Databases) or can be calculated using suitable software (Dmol^3^ or TURBOMOLE). Therefore, calculations do not require so many computational resources. The main benefit of this concept is that it takes into account acid-base interactions that favor solubility and miscibility [94]. Just et al. [95] for the first time developed group contribution set based exclusively only on pharmaceutical relevant solids to predict the solubility of the drug in polymer for solid dispersion systems prepared by hot-melt extrusion. Although in the initial study, this group contribution system showed improved prediction ability of drug solubility compared to existing methods, further experiments are necessary to enrich group contribution tables with more data as well as to validate obtained methods on additional experimental data.

Solubility parameters are used as a simple screening tool for miscibility evaluation in the early solid dispersion formulations development. Simply, values of the solubility parameters of drug and polymer should be close to each other, if the drug and polymer are miscible. This means that energy released due to cohesive interactions between like molecules is counterbalanced by adhesive interactions between unlike molecules [97,98]. While the difference in the solubility parameters (Δ*δ*) in the case of Hildebrand solubility parameters is easily calculated by subtracting *δ* of the drug from *δ* of polymer, for Hansen solubility parameters difference is calculated as the Euclidean distance according to the following equation:

(23) $$\Delta \delta =\sqrt{{\left(\delta_{d1}-\delta_{d2}\right)}^{2}+{\left(\delta_{p1}-\delta_{p2}\right)}^{2}+{\left(\delta_{h1}-\delta_{h2}\right)}^{2}}$$

where subscripts 1 and 2 denote drug and polymer, respectively.

Bagley et al. [99] considered the effects of *δ_d_* and *δ_p_* as similar and combined them into single parameter *δ_v_*, while the effect of *δ_h_* is considered as different. After applying this transformation, the difference between Hansen solubility parameters of drug and polymer can be evaluated on a two-dimensional plot with *δ_v_* and *δ_h_* on the axes. Since Bagley’s plot shows superior performances over three-dimensional Hansen plot in locating regions where the polymer is soluble in solvents, this plot should be preferably used in evaluating miscibility between drug and polymer. Although the difference between solubility parameters of drug and polymer should be small if components are miscible, it is difficult to establish a threshold for Δ*δ* value below which components are considered as miscible. According to Greenhalgh et al. [100], if Δ*δ <* 7.0 MPa^1/2^, components are likely to be miscible, while if Δ*δ >* 10.0 MPa^1/2^, components are like to be immiscible. In their study, Forster et al. [101] suggested more rigorous criteria, which predict the formation of solid solution in the cases where Δ*δ <* 2.0 MPa^1/2^, while immiscibility is predicted for systems with Δ*δ >* 10.0 MPa^1/2^. For drug-polymer systems with Δ*δ* between 5.0 and 10.0 MPa^1/2^, it is difficult to make a reliable conclusion of whether the system is miscible or immiscible. Further melt extrusion experiments showed that this system successfully predicts whether a system is miscible or not in the cases where Δ*δ* < 2.0 MPa^1/2^ and Δ*δ* > 10.0 MPa^1/2^.

Solubility parameters have been extensively used as the screening tool to get some information regarding drug-polymer miscibility, alone or more commonly in conjunction with thermodynamic modeling [20,38,98,102,103,104,105]. Although this method is more or less successful to distinguish between drug-polymer miscible pairs from those pairs where miscibility problems may occur, the application of this method alone is not highly reliable and can give misleading results. When applying solubility parameters for the evaluation of drug-polymer miscibility, one should be aware of some limitations of this concept. This is a purely theoretical concept, wherein drug-polymer interactions are based on chemical similarity, and it takes into account only enthalpic contribution to drug-polymer mixing. For further thermodynamic interpretation of the drug-polymer mixing, this method is used in conjunction with Florry-Huggins thermodynamic modeling, which has been explained in the further text. One of the main limitations of this method is that it is qualitative and does not provide any quantitative information regarding drug-polymer miscibility as well as the physical state of the API (active pharmaceutical ingredient) after mixing with the polymer [98]. It should also bear in mind that the application of different group contribution methods will inevitably give different values of solubility parameters and even the same group contribution method will give a different result if the structure of the molecule is divided in different ways. However, solubility parameters can still be considered as a useful screening tool in the early formulation development, wherein bringing of any conclusions regarding drug-polymer miscibility/immiscibility requires further application of experimental techniques and thermodynamic modeling.

### 3.2. Thermodynamic Modeling

Although analytical methods, described in Section 2, are capable of more or less accurate determination whether the drug and polymer form single-phase system or not, obtained results are valid only at the moment of analysis. It is more important to get insight into thermodynamics of the drug-polymer mixing since this will enable identification of drug and polymer composition ranges, where the formation of single-phase system is more likely to occur as well as identification of potential destabilization mechanisms. Additionally, miscibility or solubility of the drug in the polymeric matrix, below polymer’s *T_g_*, can be only estimated by model prediction due to very slow system equilibration, which gives more significance to this approach.

It has been recognized that thermodynamic models describing the mixing of small molecules with a solvent are not suitable to describe mixing between the drug and polymer since they do not take into account large volume differences between polymer and drug molecules [11]. The suitable model should relate free energy change upon mixing to volume fractions, rather than mole fractions of the components since entropic contribution to the free energy change is significantly reduced by mixing a large molecular weight molecule with a small molecular weight molecule. Flory-Huggins lattice theory that has been originally developed to describe mixing in the polymer-polymer and polymer-solvent blends is further applied to describe mixing between the drug and polymer by considering drug molecule analogous to the solvent molecule. According to this theory, free energy change upon mixing (Δ*G_mix_*) of the drug and polymer is given by the following equation [11]:

(24) $$\frac{\Delta G_{mix}}{RT}=n_{drug}ln\Phi_{drug}+n_{polymer}ln\Phi_{polymer}+n_{drug}\Phi_{polymer}\chi$$

where *n_drug_* is the number of moles of the drug, *n_polymer_* is the number of moles of polymer, Φ*_drug_* is the volume fraction of the drug, Φ*_polymer_* is the volume fraction of the polymer, *R* is the gas constant, *T* is the absolute temperature, and *χ* is Flory-Huggins interaction parameter.

The first two terms on the right side of the Equation (24) describe entropic contribution to the free energy of mixing, which always favors mixing, since mixing of two components increases system disorder. Since entropic contribution to the free energy of mixing in drug-polymer systems is much lower compared to the small molecule-solvent system, enthalpy of mixing will mainly determine whether drug-polymer mixing will occur spontaneously (Δ*G_mix_ < 0*) or not (Δ*G_mix_ > 0*). The contribution of the enthalpy of mixing to the overall free energy of mixing is determined by the sign and magnitude of the Flory-Huggins interaction parameter χ, which reflects the strength of drug-polymer adhesive interactions relative to cohesive interactions between drug-drug and polymer-polymer pairs. Negative values of χ indicate stronger adhesive interactions, which facilitate drug-polymer mixing, as a result of negative Δ*G_mix_*. In the case of positive χ, which indicates stronger cohesive interactions, drug-polymer mixing will be thermodynamically favored only if the entropic contribution to Δ*G_mix_* overcomes unfavorable enthalpy of mixing and gives negative overall free energy of mixing [11,106,107]. Since the strength of adhesive interactions between the drug and polymer is determined by their chemical structures, shifting from one to other chemical class of polymers is a better approach to achieve miscibility than shifting from higher to lower molecular weight grade of the same polymer [11]. Although values of χ can be determined by different approaches, the most common way to determine χ in drug-polymer systems is a melting point depression method. In this method, physical mixtures of drug and polymer of various compositions are subjected to DSC scan and, if the system is miscible, drug melting point should be reduced in the mixture compared to the melting point of the pure drug. Melting of the drug occurs at a temperature where the chemical potential of the crystalline drug becomes equal to the chemical potential of the molten drug. Mixing of drug with polymer will reduce drug chemical potential and, therefore, the melting of the drug will occur at a lower temperature, compared to the pure drug. If the drug and polymer are immiscible, the melting of the drug will be unaltered in the presence of polymer [14,15,20]. The relationship between the melting point depression upon drug-polymer mixing and χ is given by the following equation [11]:

(25) $$\left(\frac{1}{T_{M}^{mix}}-\frac{1}{T_{M}^{pure}}\right)=\frac{-R}{\Delta H_{fus}}\left[ln\Phi_{drug}+\left(1-\frac{1}{m}\right)\Phi_{polymer}+\chi \Phi_{polymer}^{2}\right]$$

where $T_{M}^{mix}$ is the melting temperature of the drug in the presence of the polymer, $T_{M}^{pure}$ is the melting temperature of the pure drug, Δ*H_fus_* is the heat of fusion of the pure drug, and *m* is the ratio of the volume of the polymer to that of the lattice site (defined as the volume of the drug molecule). This equation is further rearranged to give the plot of

(26) $$\left(\frac{1}{T_{M}^{mix}}-\frac{1}{T_{M}^{pure}}\right)\times \left(\frac{\Delta H_{fus}}{-R}\right)-\ln\left(\Phi_{drug}\right)-\left(1-\frac{1}{m}\right)\Phi_{polymer}\;vs.\;\Phi_{polymer}^{2}$$

which exhibits linear relationship within the low polymer concentrations with the slope equal to χ. When using melting point depression method to estimate χ, one should be aware of some limitations of this approach. Firstly, *T_g_* of polymer should be sufficiently below drug melting temperature because the crystalline drug should interact with the polymer in a supercooled liquid state sufficiently long before it starts to melt. Additionally, linearity in the plot used to calculate χ is limited to low polymer concentrations. Although markedly different values of χ are obtained if used onset, midpoint, or offset of drug melting peak in the DSC scan as a drug melting temperature [14], there is no consensus in the literature regarding which value should be used. Marsac et al. [14] and Paudel et al. [15] proposed that offset of melting endotherm should be used since it represents the melting of the final composition after the occurrence of mixing. However, in most of the studies, onset values have been used [17,19,20,104], where the low heating rate is used to facilitate mixing within the experimental time scale. Calculated χ enables estimation of the free energy changes upon drug-polymer mixing (Δ*G_mix_*) as a function of the drug weight fraction, according to the Equation (24). However, values of χ obtained by the melting point depression method are valid only at drug melting temperature and cannot be used to predict drug-polymer miscibility at lower temperatures. It has been shown that χ varies with temperatures and composition. Since the effect of the composition on χ is considered as negligible, compared to the effect of temperature, the temperature dependence of χ can be expressed using the following simplified equation [12,18]:

(27) $$\chi =A+\frac{B}{T}$$

where *A* is referred to as the non-combinatorial entropic contribution to χ, while *B*/*T* is the enthalpic contribution [108]. By measuring melting points of the drug in mixtures of different compositions, different values of χ can be obtained, and by plotting these values as a function of corresponding temperatures, parameters *A* and *B* can be obtained. This enables calculation of χ at any temperature and prediction of Δ*G_mix_* as a function of temperature and composition. Solubility parameters can be also used to calculate χ, according to Equation (28):

(28) $$\chi =\frac{V_{site}}{RT}{\left(\delta_{drug}-\delta_{polymer}\right)}^{2}$$

where *V_site_* is the volume of the hypothetical lattice (approximated as the volume of the drug).

Since χ calculated in this way reflects drug-polymer interactions at 25 °C, this value can be additionally used to estimate temperature dependence of χ, according to Equation (27) [17,20]. Although the calculation of χ using solubility parameters is the simplest approach and does not require any experiment, it has been shown that obtained χ can fail to predict drug-polymer miscibility [11]. This probably comes from the inherent limitation of solubility parameters approach in systems with pronounced specific intermolecular interactions. An additional limitation of this approach is that it does not take into account possible exothermic mixing since calculated χ is always positive [15].

Besides the estimation of the drug-polymer miscibility, thermodynamic modeling is used to estimate drug solubility in polymers used for solid dispersions preparation. Only if drug concentration in solid dispersion is below the solubility limit, physical stability of this system is guaranteed without the tendency of drug toward crystallization. Therefore, estimation of the drug solubility in the polymeric matrix is of particular importance since it is an indicator of the degree of supersaturation, which determines driving force for drug crystallization. If the amount of drug in solid dispersion is above its solubility in the polymer, but below the miscibility limit, the system is considered as metastable and is stabilized against crystallization through intimate mixing with polymer, unless large fluctuations of temperature and/or composition occur, which makes favorable conditions for crystallization [12]. Marsac et al. [14] developed a model for the estimation of the solubility of the drug in polymers by using the measured drug solubility in low molecular weight analog of the polymer. The solubility of the drug in the low molecular weight analog is given by the following equation [14]:

(29) $$ln\;x_{drug}\gamma_{drug}=\frac{-\Delta G_{fus}}{RT}=-\frac{\Delta H_{fus}\;T_{m}}{RT}\left[1-\frac{T}{T_{m}}\right]-\frac{1}{RT}\int_{T_{m}}^{T}\Delta C_{p}dT+\frac{1}{R}\int_{T_{m}}^{T}\frac{\Delta C_{p}}{T}dt$$

where *γ_drug_* is the activity coefficient of the drug in the mixture at the solubility limit, *x_drug_* is the mole fraction of dissolved drug, Δ*G_fus_* is the free energy difference between supercooled liquid and crystal, *T* is the temperature of interest, *T_m_* is melting temperature, *R* is universal gas constant, Δ*H_fus_* is heat of fusion, and Δ*C_p_* is the capacity difference between the liquid and crystal.

By considering that Δ*C_p_* does not change significantly in the temperature range of interest, Equation (29) can be rewritten into the following form [109]:

(30) $$ln\;x_{drug}\gamma_{drug}=-\frac{\Delta H_{fus}}{RT}\left[1-\frac{T}{T_{m}}\right]-\frac{\Delta C_{p}}{R}\left[1-\frac{T_{m}}{T}+ln\;\left(\frac{T_{m}}{T}\right)\right]$$

Since all parameters in the Equations (29) and (30) can be easily experimentally determined, except activity coefficient (*γ_drug_*), calculation of *γ_drug_* is a necessary prerequisite to determine drug solubility. By considering ideal mixing (*γ_drug_* = 1), ideal solubility of the drug in the low molecular weight analog can be calculated from Equation (30). The ratio of ideal solubility to experimentally determined the solubility of the drug in the low molecular weight analog of polymer gives *γ_drug_* in low molecular weight analog of the polymer (*γ_drug_^LMW^*). The activity coefficient of the drug in the polymer (*γ_drug_^polymer^*) is considered equal to *γ_drug_^LMW^* after the addition of correction factor to reduce the entropy of mixing of the drug in polymer compared to the low molecular weight analog:

(31) $$ln\;\gamma_{drug}^{polymer}=\frac{MV_{drug}}{MV_{lattice}}\left[\frac{1}{m_{drug}}ln\frac{\Phi_{drug}}{x_{drug}}+\left(\frac{1}{m_{drug}}-\frac{1}{m_{polymer}}\right)\Phi_{polymer}\right]+ln\;\gamma_{drug}^{LMW}$$

where *MV_lattice_* is lattice molecular volume (in this case, defined as volume of low molecular weight analog of polymer), *MV_drug_* is drug molecular volume, *m_polymer_* is the ratio of the volume of the polymer to that of the lattice site, *m_drug_* is the ratio of the volume of the drug to the lattice site [14].

Calculated *γ_drug_^polymer^* can be further used to calculate χ, as an alternative approach compared to commonly used melting point depression method [14]:

(32) $$ln\;\gamma_{drug}^{polymer}=\frac{MV_{drug}}{MV_{lattice}}\left[\frac{1}{m_{drug}}ln\frac{\Phi_{drug}}{x_{drug}}+\left(\frac{1}{m_{drug}}-\frac{1}{m_{polymer}}\right)\;\Phi_{polymer}+\chi \;\Phi_{polymer}^{2}\right]$$

Marsac et al. [14] calculated the solubility of several drugs in different grades of PVP using solubility data in the 1-ethyl-2-pyrrolidone as the low molecular weight analog of PVP. Obtained results show significantly reduced solubility in polymer due to reduced entropy of mixing up to the certain molecular weight of the polymer after which solubility is only slightly changed. Additionally, χ calculated using this approach is in agreement with those obtained by melting point depression method. The same method has been successfully used by Paudel et al. [15] to predict the solubility of naproxen in different grades of PVP using measured solubility of naproxen in N-methylpyrrolidone as a low molecular weight analog of PVP. Although this approach is quite simple and does not require so many experimental resources, it assumes that interactions between the drug and polymer are the same as between the drug and the low molecular weight analog (i.e., χ is the same in both cases) and is applicable only for polymers with available low molecular weight analog in the liquid state. Djuris et al. [19], in their study, used Hansen solubility parameters to calculate the activity coefficient of carabamazepine in polyethyleneglycol-polyvinylcaprolactam-polyvinyl acetate grafted copolymer (Soluplus^®^) according to the following equation:

(33) $$ln\;\gamma_{drug}=\frac{V_{drug}}{RT}\left[{\left(\delta_{d}^{drug}-{\bar{\delta }}_{d}\right)}^{2}+0.25\left({\left(\delta_{p}^{drug}-{\bar{\delta }}_{p}\right)}^{2}+{\left(\delta_{h}^{drug}-{\bar{\delta }}_{h}\right)}^{2}\right)\right]+ln\frac{V_{drug}}{\bar{V}}+1-\frac{V_{drug}}{\bar{V}}$$

(34) $$\bar{\delta }=\sum_{k=1}^{n}\Phi_{k}\delta_{k}$$

where $\bar{\delta }$ is the molar volume-weighted solubility parameter, and $\bar{V}$ is the mixture volume, where the subscript k denotes the different components of the mixture.

Obtained activity coefficient shows strong composition dependence and can be used to estimate mole fraction of dissolved drug within the polymeric matrix using either Equation (30) or Equation (32). Results obtained via both ways are in close agreement and show that the amount of carbamazepine that can be molecularly dispersed in the Soluplus^®^ matrix is limited to below 5% (*w*/*w*) [19]. 

Prudic et al. used thermodynamic modeling based on perturbed-chain statistical associating fluid theory (PC-SAFT) to estimate drug solubility in polymer. According to this theory, each molecule is described as a chain composed of spherical segments that can interact with segments of other molecules through different types of interactions. In PC-SAFT model, the residual Helmholtz energy *a^res^* of a system containing drug and polymer is calculated as the sum of reference hard-chain contribution accounting for repulsive interactions between molecules (*a^hc^*), a dispersion contribution accounting for van der Waals attraction forces (*a^disp^*), and a contribution caused by association via hydrogen bonds (*a^assoc^*) [110]:

(35) $$a^{res}=a^{hc}+a^{disp}+a^{assoc}$$

Segment number *m_i_^seg^* and segment diameter *σ_i_* are used to calculate hard-chain contribution, while the contribution from the van der Waals attraction forces between segments (*a^disp^*) is calculated using dispersion-energy parameter *u_i_/k_b_* (*u_i_*—dispersion energy, *k_b_*—the Boltzmann constant). Additionally, for drugs and polymers capable of hydrogen bonds formation, it is necessary to calculate a contribution caused by association via hydrogen bonds (*a^assoc^*). The calculation of this term requires definition of the number of association sites (electron acceptors and donors) *N_i_^assoc^*, defined based on the molecule’s chemical structure, the association-energy parameter *ε^AiBi^/k_B_* (related to the strength of association), and the association-volume parameter *κ^AiBi^* (related to the distance between two molecules necessary to form a hydrogen bond). These pure-component parameters of drugs and polymers, required for the calculation of the residual Helmholtz energy, are usually determined by fitting to experimental solubility data of these components in organic solvents. The calculation of the residual Helmholtz energy of the system is described in detail elsewhere [110].

The activity coefficient of the drug in the liquid drug/polymer phase (*γ_i_^L^*), required for the calculation of solid-liquid equilibrium curve (Equation (30)), can be calculated by PC-SAFT method using the following equations [111]:

(36) $$\gamma_{i}^{L}=\frac{\phi_{i}^{L}}{\phi_{0i}^{L}}$$

(37) $$ln\;\phi_{i}^{L}=\frac{\mu_{i}^{res}}{k_{B}T}-ln\;Z$$

(38) $$Z=\frac{pV}{k_{B}N_{A}T}$$

(39) $$\frac{\mu_{i}^{res}}{k_{B}T}=\frac{a^{res}}{k_{B}T}+Z-1+\left(\frac{\partial \left(a^{res}/k_{B}T\right)}{\partial x_{i}}\right)-\sum_{j=1}^{N}x_{j}\left(\frac{\partial \left(a^{res}/k_{B}T\right)}{\partial x_{j}}\right)$$

where $\phi_{i}^{L}$ is fugacity coefficient of component *i* in the mixture, $\phi_{0i}^{L}$ is fugacity coefficient of the pure component, $\mu_{i}^{res}$ is residual chemical potential, *Z* is compressibility factor, *p* is system pressure, and *N_A_* is the Avogadro number.

PC-SAFT is successfully used to predict the solubility of artemisinin and indomethacin in PEGs of different molecular weights as a function of temperature and predicted results are in close agreement with experimentally obtained data [110]. This approach is also used to predict long-term stability of the drug in both binary and ternary solid dispersions and evaluate the impact of relative humidity on drug recrystallization and amorphous-amorphous phase separation [110,111,112,113,114]. The solubility of acetaminophen in PVP K25 and PVP VA64 and the impact of relative humidity on the solubility are predicted by PC-SAFT and Flory-Huggins modeling and further used as an indicator of long-term stability of acetaminophen solid dispersions in these two polymers. Obtained results show that the PC-SAFT method gives more accurate prediction and can better differentiate whether solid dispersions remain stable or undergo recrystallization under elevated humidity [111]. Advantage of PC-SAFT method is that each component is characterized with parameters that are physically meaningful and do not depend on the temperature, component molecular weight, concentration, etc. Additionally, this method takes into account different types of interactions in the system, such as association (hydrogen bonding) and ionic and polar interactions between the compounds [115]. It has been also demonstrated that the PC-SAFT method enables accurate prediction of the drug solubility in copolymers if the drug solubility in the respective homopolymers is known [115]. Although the PC-SAFT method requires less experimental work than Flory-Huggins method, it requires more complicated calculations. However, once determined, parameters of the pure component can be further used for other systems, which contains that component, so it is expected that this method will be more frequently used in the future upon an increase in the availability of necessary component parameters in the literature.

#### Construction of the Phase Diagram

As stated above, the calculation of χ at different temperatures, using Equation (27), enables prediction of the free energy change upon drug-polymer mixing as a function of both temperature and composition. As long as Δ*G_mix_ < 0* and Δ*G_mix_* vs. composition curve is concave up, the formation of the single-phase system occurs since free energy of the mixture is lower than the free energy of the two-phase system. Phase separation can occur only if the single-phase system can lower its free energy by separating into two phases [104]. Determination of Δ*G_mix_* vs. composition curve at different temperatures is a necessary prerequisite to constructing temperature vs. composition phase diagram, which shows phase behavior of drug-polymer mixture, and differentiate regions of stability, metastability, and instability. Phase behavior has not been studied for so many drug-polymer systems, and available studies describe the different methodology to construct a drug-polymer phase diagram. Phase diagrams have been described for solid dispersions of dipyridamole and cinnarizine in PVP and polyacrylic acid (PAA) [20], cinarizine in Soluplus^®^ [8], indomethacin in polyvinylpyrrolidone-vinyl acetate (PVP VA) copolymer [17], itraconazole in HPMC [106], felodipine in HPMCAS HF grade and Soluplus^®^ [18], PAA [16] and different grades of PVP [103], aceclofenac in Soluplus^®^ [104], naproxen and acetaminophen in HPMCAS, PVP K25 and PVP VA64 [112], and binary polymeric blends containing HPMCAS and either PVP K25 or PVP VA64 [113]. Typical phase diagram includes a solid-liquid phase transition curve, amorphous phase separation curve, and glass transition curve. In their work, Tian et al. [8] calculated drug solubility curve for cinarizine in Soluplus^®^ using solid-liquid equilibrium equation, which considers that the polymer behaves as a solvent for a crystalline drug:

(40) $$ln\;x_{drug}=\frac{\Delta H_{fus}}{RT_{m}}\left(1-\frac{T_{m}}{T}\right)-ln\;\gamma_{drug}$$

Lehmkemper et al. suggested that solubility of the drug in polymer should be assessed using Equation (30), which includes Δ*C_p_* term and, therefore, should give more accurate results [111]. Activity coefficient, required for the calculation of the solubility curve, can be calculated using Hansen solubility parameters, according to above-mentioned Equation (33). Solid-liquid phase transition curve can be also calculated using melting point depression approach (Equation (25)), considering Flory-Huggins interaction parameter calculated at different temperatures, as described by Lin et al. [16] and Tian et al. [18]. The PC-SAFT method also enables the prediction of the solid-liquid phase transition curve in the drug-polymer systems and, in some cases, gives more accurate results compared to Flory-Huggins modeling [110,111].

While the solid-liquid phase transition curve describes the solubility limit of the drug in the polymer, miscibility limit of two phases, i.e., the tendency towards amorphous-amorphous phase separation is described by binodal and spinodal curves. The binodal curve is determined by the common tangent rule to free energy vs. composition curve, where the first derivative of this curve is set to zero [17,108]. Above this curve, single-phase amorphous system is formed, while in the region below binodal and above the spinodal curve, the system is metastable, i.e., large composition fluctuation is necessary to induce phase separation [8]. Phase separation process between binodal and spinodal curves can occur via nucleation and growth mechanisms, only if the significant energetic barrier is overcome [18]. The spinodal curve is obtained by setting the second derivative of free energy vs. composition curve to zero according to the following equation [17,18,20]:

(41) $$\frac{1}{\Phi_{drug}}+\frac{1}{m_{polymer}}\frac{1}{\left(1-\Phi_{drug}\right)}-2\chi_{drug-polymer}=0$$

After the determination of the temperature dependence of χ, Equation (41) can be transformed in the following form, which enables the construction of the spinodal curve on the temperature vs. composition phase diagram [16,104]:

(42) $$T_{s}=\frac{2B}{\frac{1}{\Phi_{drug}}+\frac{1}{m\;(1-\Phi_{drug})}-2A}$$

The spinodal curve represents phase boundary between metastable and unstable region, and, below this curve, spontaneous (barrier-free) phase separation into drug-rich and polymer-rich regions occurs, which is often denoted as spinodal decomposition. The glass transition curve is an important part of the temperature composition phase diagram as an indicator of system kinetic stabilization. Although solubility and miscibility limits can be exceeded, phase separation and crystallization can be avoided, through kinetic stabilization of the system below *T_g_* of the mixture. Polymers with high *T_g_* are preferred in the formulation of solid dispersions due to increasing *T_g_* of the mixture, which is denoted as an antiplasticization effect. Below *T_g_*, viscosity drastically increases and molecular mobility decreases, which altogether hinders crystallization of drug molecules. It is often reported that molecular mobility can be neglected at temperatures more than 50 °C below the mixture *T_g_* [116]. Therefore, although the system is thermodynamically not stable, it can be kinetically stabilized during product shelf life. Kinetic stabilization is particularly important when using techniques for solid dispersions preparation where materials are processed under non-ambient conditions, such as hot-melt extrusion. In this technique, mixing of the molten drug and polymer above polymer *T_g_* and/or dissolving of the crystalline drug within the polymer above its *T_g_* is facilitated by high processing temperature and high shear stress applied by mixing elements. During cooling to room temperature, homogeneously mixed or dissolved drug can be kinetically frozen in that state for a sufficiently long time, although above solubility and miscibility limit for a particular temperature. After construction, the phase diagram should be validated. This is commonly performed by preparing the solid dispersions of different composition and evaluating drug crystallinity by XRPD as well as the presence of phase separation and/or drug recrystallization by DSC or similar thermal analysis techniques [17,18,104].

**Figure 2:**
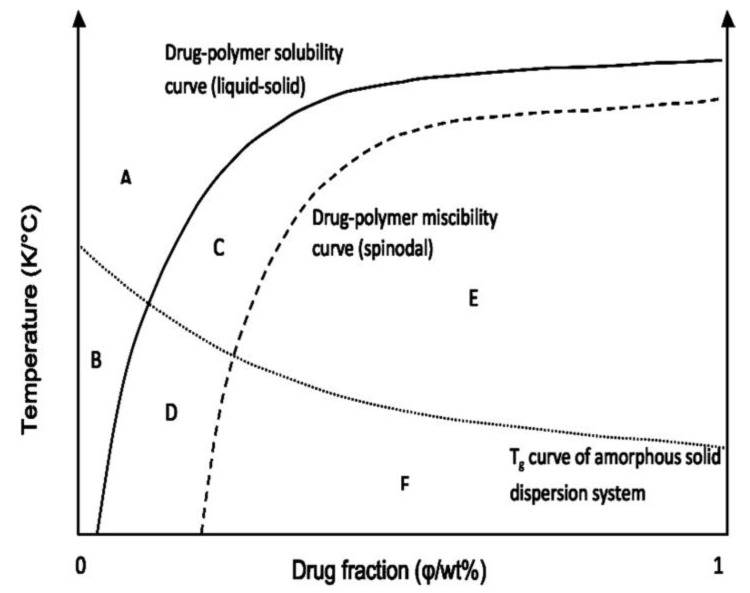
Typical temperature vs. composition phase diagram for the binary drug-polymer system (Reproduced with permission from Tian et al., 2013). [18]. Copyright (2013) American Chemical Society.

Due to simplicity and not a straightforward calculation of binodal curve, the phase diagram is often represented with solid-liquid phase transition curve (solubility curve), spinodal (miscibility) curve, and glass transition curve, as shown in Figure 2 [18]. Above the solubility curve (Zone A and B), the drug is dissolved in the polymer and is stable to temperature and concentration fluctuations. Even if crystallization starts, the thermodynamic driving force in this region is to dissolve the crystalline drug in the polymer. Although this should be the most desired region in the development of solid dispersions formulations, the solubility of the drug is usually too low and limits practical formulation development only to very low dose drugs. Since the amorphous drug has a higher chemical potential compared to the crystalline drug, the miscibility of the amorphous drug with the corresponding polymer is much higher than the solubility of the crystalline drug in the polymer. Therefore, the miscibility boundary is more relevant to the solid dispersion formulation development. Below this boundary line (zones E and F), the system is thermodynamically unstable, and spontaneous phase separation will occur. Only within the zone F, the system can be stabilized kinetically, if stored at temperatures sufficiently below *T_g_*. Above miscibility and below solubility curve (zones C and D), the system is supersaturated with respect to drug solubility but is stabilized through mixing with polymer, and phase separation requires certain activation energy. In the zone D, the system is additionally kinetically stabilized due to reduced molecular mobility below *T_g_*.

### 3.3. Computational Modeling and Simulations

Following the advances in informatics technologies, which led to increased computing power and speed, together with the high availability of reliable free or affordable proprietary molecular modeling software capable of handling large systems, the simulation of the solid-state has become possible to a very satisfactory degree of precision and time scale. Recent applications of molecular modeling relevant to the formulation of poorly soluble drugs are numerous and focusing on various aspects of the solid-state.

Specifically, molecular dynamics (MD) simulations, a highly powerful molecular modeling technique for the study of physical movement of atoms and molecules by numerically solving Newton’s equation of motion, is gaining increased attention in the recent years. In MD simulations, interatomic potentials or molecular mechanics force fields are used to calculate the potential energies and forces occurring between the simulated atom particles [117,118]. In brief, during an MD simulation, the components are initially identified (molecules and concentrations), and the interaction functions (or else “force fields”) are set. Then, after setting the desired thermodynamic conditions (i.e., density, pressure, and temperature), the initial positions of molecules are defined, and the velocities of atoms are randomly assigned. Finally, the simulation starts, and, depending on the property under investigation, the thermodynamic parameters may change or fluctuate until the whole system equilibrates in a given set of conditions, i.e., mimicking the procedure of macroscopic equilibration process in a real laboratory experiment [119].

Based on the satisfactory degree of precision and the rather simple and easy to interpret theoretical background, MD simulations have gained increased attention regarding the in-depth evaluation of pharmaceutical solid-state processes. In recent years, such attempts include the simulation of API amorphous state [120], API crystallization processes from supersaturated solutions [121], API—water interactions [122], and API—matrix carrier interactions [123]. Based on the required level of detail, simulations may be performed from picoseconds up to several hundred nanoseconds.

As noted from a recent expert review published by Edueng et al. [124], although the use of MD simulations exhibits extremely promising results in characterizing both pure API amorphous state and API—carrier molecular interactions, this methodology is still only used relatively sporadically. This may be attributed probably to the improper realization or training of the scientists working in the field. Therefore, to alleviate the poor perception of scientists on the subject, it is attempted in the following section, to present a detailed overview of the currently available advanced computational models, used specifically for the estimation of miscibility and molecular interactions (an indirect indication of miscibility) occurring between solid dispersion components.

In this direction, Gupta et al. have performed MD simulations to predict the miscibility of pharmaceutical compounds [120]. Specifically, the authors developed a computational model (verified experimentally via thermoanalytical techniques), which can predict the miscibility of indomethacin in several carriers (polyethylene oxide, glucose, and sucrose). In all applied MD simulations, the COMPASS (Condensed-phase Optimized Molecular Potentials for Atomistic Simulation Studies) force field is used, which is parameterized based on ab initio quantum mechanics calculations. According to the authors, after an initial energy minimization step, the MD simulations are carried out in two phases: (1) the equilibration run, where the amorphous cells are allowed to relax for 2 ns under isothermal (NVT) or isobaric-isothermal conditions (NPT-NVT) at 298 K and (2) the production run where the equilibrated structure is processed via the NVT ensemble for 200 to 500 ps at 298 K with a time step of 1 fs. The Andersen thermostat and barostat are used to maintain the temperature and pressure stable, respectively. The non-bonded van der Waals and electrostatic interactions are truncated using the group-based cut-off distance of 1.25 nm. Trajectory frames are captured during the production run, and the data from the final 50 ps are used for computing the Cohesive Energy Density (CED) and solubility parameter (δ). Results show that the employed MD simulations can predict successfully indomethacin miscibility with polyethylene oxide and immiscibility with sucrose and glucose.

In another paper, the same group of authors used MD simulations for predicting glass transition temperature and plasticization effect in amorphous pharmaceuticals [122]. Amorphous sucrose (widely used as a carrier in the preparation of solid dispersions) and water are selected as model compound and plasticizer, respectively. As in their previous work, MD simulations are performed using the COMPASS force field and isothermal-isobaric ensembles in two steps (equilibration and production phase). In this study, to predict *T*_g_, the authors allowed the system to stepwise cooling from 440 K to 265 K at 5 K intervals by using the final structure from each MD run as the starting structure for the subsequent run. The density is measured at every picosecond interval during the last 50 ps run at each temperature step of the production run, and the average density values are used to calculate the specific volume. Specific volume vs. temperature plots is used to estimate MD-based *T_g_* value for amorphous sucrose containing 0%, 3%, and 5% *w*/*w* water, respectively, which are in reasonable agreement with the experimental values reported in the literature. Additionally, radial distribution function analysis of the MD trajectories reveals strong hydrogen bond interactions between sucrose hydroxyl oxygen and water oxygen.

In another study, Maus et al. used MD simulations to predict miscibility and *T_g_* for pharmaceutical solid dispersion systems prepared by a melt-based method, such as hot-melt extrusion [125]. Different mixtures containing theophylline or ibuprofen and water-soluble (triethyl citrate) or water-insoluble (acetyl tributyl citrate or dibutyl sebacate) plasticizers dissolved or dispersed in a cationic polymethacrylate matrix carrier have been evaluated. Initially, for the MD simulations, cubic simulation boxes (with periodic boundary conditions in all directions) are constructed (side length of ca. 4 nm). Then, after appropriate energy minimization, the structures are left to relax for 2 ns under NPT conditions at ambient conditions, to obtain a well-relaxed start structure with the correct density using the Andersen thermostat and barostat, at a time step of 1 fs. Afterward, a 200 ps run at constant volume and temperature (NVT) is carried out (100 ps for equilibration and 100 ps for data sampling). The cohesive energy (*E_coh_*) is averaged over this latter period, and the corresponding cohesive energy density is calculated by dividing it through the volume (*V*) of the simulation cell (*E_coh_*/*V*). In all MD simulations, a cut-off distance of 1.25 nm with a spline switching function is applied for the Coulomb and van der Waals interactions using charge groups to prevent dipoles from being artificially split. Atomic charges and interactions between atoms and molecules are accounted for by the use of the COMPASS force field. For *T_g_* evaluation, the specific volume vs temperature diagrams are constructed by relaxing the systems for 2 ns under NPT conditions at a temperature of approximately 100 K above the supposed *T_g,_* followed by a cooling process with a stepwise of 10 K until the temperature is ~100 K below *T_g_*. Results show that the use of Hilderbrand’s solubility parameter estimated via MD calculations leads to an incomplete picture of the system’s miscibility, while better results are obtained when MD-based Gibbs free energy is used. Additionally, the correlation of the simulated *T_g_* with the experimentally determined values reflects the different solubility behaviors of the plasticizers studied (less miscible plasticizers show a higher deviation from the experimental *T_g_*).

In a similar work, published by Macháčková et al. [126], MD simulations are employed evaluate miscibility of cyclosporine-A in six biodegradable polymers, namely l-polylactide, d-polylactide, chitosan, polyglycolic acid, PEG, and cellulose [126]. All prepared models are optimized using PCFF (Polymer Consistent Force Field) force field, while smart algorithm (a cascade of steepest descent, conjugate gradient, and quasi-Newton methods) with 50,000 steps is used for geometry optimization, while atomic charges are assigned by a PCFF force field. For MD simulations, periodic boundary conditions are employed under NPT dynamics with Nose thermostat and Berendsen barostat for 1.5 ns. The Flory-Huggins interaction parameter, χ, describing API-polymer miscibility is calculated based on the mixing energy (*E_mix_*) representing the difference in free energy between the mixture and the sum of pure state energy of both components (API and polymer). With the present work, the author revealed that MD-simulations could be a powerful tool for predicting component miscibility. Specifically, results show that miscibility is dependent on chain length and this dependence is more noticeable for flexible chains, while the best miscibility is strongly correlated with the polymer-drug interaction energy and with the number of hydrogen bonds between polymer and drug molecule. Additionally, MD simulations can show that the two polymers (polycellulose and polychitosan), with the best miscibility and the highest polymer-drug adhesion, exhibit surprisingly higher rigidity.

Barmpalexis et al. [127] have also used MD simulations to study the miscibility of three commonly used plasticizers (namely, citric acid, triethyl citrate, and PEG) with Soluplus^®^, a widely used polymeric matrix in hot-melt extrusion processes [127]. MD simulations are performed using pcff_d force field under NPT with a cut-off radius of 7 Å, spline distance of 1 Å, Berendsen thermostat, variable volume and shape option, and 1 fs time step. The cohesive energy (*E_coh_*, i.e., the measure of the intermolecular forces acting between molecules) is calculated after 2 ns structure relaxation process and another 400 ps run under NVT, to calculate the Hildebrand solubility parameter by dividing the square root of *E_coh_* with square root of simulation volume (*V*) (Equation (9)). Simulations show miscibility only in the case of Soluplus^®^ and PEG, a result that is verified experimentally by the presence of significant molecular interactions between the two components.

In another paper, the same group of authors tried to expand their previously developed molecular model (polymer-plasticizer matrix system) by including two BCS (Biopharmaceutics Classification System) class II model drugs (namely ibuprofen and carbamazepine) having substantially different thermal properties and glass-forming ability [123]. The same set of MD simulation parameters, as the once selected previously, are used in this attempt. Simulations results suggest that both APIs are miscible in the selected solid dispersion matrix (Soluplus^®^-PEG) verified experimentally by thermoanalytical analysis (DSC).

MD simulations (using AMBER 11 force field) are also utilized to evaluate the molecular structures of solid dispersions by the simulated annealing method, mimicking the hot-melt preparation method [128]. During the minimization procedure, the structures are subjected to 1000 steps of steepest descent minimization followed by 1000 steps of conjugate gradient minimization. After minimization, a 1 ns simulated annealing simulation, with a Langevin dynamics is used in a 2 fs time step and a cut-off of 12 Å for non-bonded interactions. During the simulated annealing procedure, the system is initially heated from 0 to 500 K in 200 ps and then is kept at temperature for 300 ps to equilibrate. Next, the system is quickly cooled down from 500 to 300 K in 100 ps and is kept at that temperature for 400 ps. The simulated annealing procedure is repeated 10 times (10 ns) for complete convergence of the systems. Based on the presented results, the authors have succeeded in developing an all-atomic MD model for the formation of solid dispersions prepared by hot-melt method and the molecular mechanisms involved during such preparations.

Finally, in a similar attempt, Xiang and Anderson [129] have also used a simulated annealing method, mimicking the hot-melt preparation in an attempt to investigate the molecular interactions occurring between indomethacin and PVP. All MD simulations are performed via Amber-ff02 force field. The prepared initial structures after energy minimization are left to equilibrate at 600 K for approximately 10 ns and then subjected to cooling dynamic runs to a final temperature of 200 K at a cooling rate of 0.03 K/ps. The newly formed glasses are used as starting configurations for prolonged aging dynamic runs (~100 ns) at 298 K and 1 bar. MD simulations suggest that the two components are miscible, a result that is verified by the formation of strong specific interactions (hydrogen bonds).

## 4. Conclusions

Evaluation of the drug-polymer miscibility and solubility of the drug in the selected polymer has become an unavoidable part in the rational design of solid dispersion formulations. There are numerous approaches to estimate the drug-polymer solubility/miscibility, which we grouped into analytical and computational methods, although this classification is rather arbitrary since computational methods in most cases use data obtained by analytical methods. However, one should be aware that each analytical technique alone has its limitations to differentiate between the drug and polymer domains, whereas computational methods have some inherent assumptions since they have not been developed specifically for the drug-polymer systems. Since the application of any of either computational or analytical methods alone provides only one part of the complete picture, the evaluation of the drug-polymer miscibility and solubility of the drug in polymer requires a multimethodological approach. Future research in this field should enable the development of a standardized methodology for the estimation of drug-polymer solubility/miscibility, which is of the utmost importance for the development of solid dispersion formulations, as well as the final dosage forms, in the pharmaceutical industry. We propose that standardized methodology should be based on the thermodynamic modeling, a coupled computational-analytical method, as this approach enables straightforward construction of temperature vs. composition phase diagram, which further serves as a guidance for formulation scientists to choose suitable polymer, drug loading, processing conditions, and storage conditions to ensure long-term stability of solid dispersion systems. Certainly, analytical methods are an inevitable part of this methodology, as it is necessary to provide experimental data for thermodynamic modeling and validation of the phase diagrams. The time-consuming calculations required for the thermodynamic modeling hinder its implementation in the pharmaceutical industry, so the development of user-friendly software solutions for thermodynamic modeling should certainly facilitate the wider application of this approach in the formulation development. Additionally, it is of particular importance to refine currently used thermodynamic models to adjust them to drug-polymer systems and to include specific drug-polymer interactions with the overall aim to improve the prediction accuracy of the models. Thorough implementation of a methodology for the assessment of drug-polymer solubility/miscibility in the development of solid dispersion formulations should bridge the gap between the great success in solid dispersion technology on the laboratory scale and difficulties for such products to reach the market.

## Figures and Tables

**Table 1:** Examples of analytical methods used for the characterization of solid dispersions.

Drug	Polymer(s) and Other Excipients	Method for Preparation of Solid Dispersions	Analytical Methods Used to Study Solubility/Miscibility	References
Albendazole	PVP	Hot-melt extrusion	DSC, XRPD, HSM, μ-CT SEM	[55]
Carbamazepine	Soluplus^®^	Hot-melt extrusion	Rheological properties, DSC, XRPD	[63]
Carbamazepine, Prednisolone	PVP, Eudragit^®^ E 100	Electrospray deposition	DSC, XRPD	[69]
Chloramphenicol	Poly(ε-caprolactone)	Film casting	DSC, XRPD, FT-IR, AFM	[70]
Diphenhydramine, Propranolol	Eudragit^®^ L 100, Eudragit^®^ L 100-55	Hot-melt extrusion	XPS, DSC, XRPD, SEM	[24]
Felodipine	PVP	Solvent evaporation	DSC, FT-IR, XRPD	[23]
	Eudragit^®^ E PO	Hot-melt extrusion	SEM, DSC, M-DSC, NMR	[26]
	Soluplus^®^, HPMCAS, PVP, Eudragit^®^ E PO, PVPVA, HPC, PAA, Na CMC, PVA, HEC	Spin coating	TASC, IR imaging	[60]
Felodipine, nifedipine, ketoconazole	PVP, PAA	Solvent evaporation	DSC, FT-IR, XRPD	[47]
Griseofulvin	HPMCAS	Co-milling	FT-IR, XRPD, DSC	[71]
Ibuprofen	Eudragit^®^ L 100	Solvent evaporation	NMR	[52]
Indomethacin	PVP	Solvent evaporation	FT-IR, FT-Raman	[33]
	Eudragit^®^ E PO	Melting or compression methods	M-DSC, rheological properties, FT-IR	[35]
Indomethacin, dextran	PVP	Solvent evaporation	XRPD, DSC	[39]
Indomethacin, nifedipine, d-mannitol	PVP, PVA	Co-milling	DSC, XRPD	[13]
Indomethacin, ursodeoxycholic acid, indapamide	PVP	Solvent evaporation	Water vapor absorption studies	[62]
Itraconazole	PEG and HPMC	Solvent evaporation (spray drying)	M-DSC, XRPD	[65]
	HPMCAS and Soluplus^®^	Film casting	XRPD, DSC, PLM	[66]
	HPMCP, Soluplus^®^, PVPVA 64, Eudragit^®^ E PO			[67]
	HPMCAS with the addition of Poloxamer 188, Poloxamer 407, or TPGS	Film casting and hot-melt extrusion	DSC, XRPD	[72]
Lacidipine	PVP K30, PVP VA64, Soluplus^®^	Hot-melt extrusion	XRPD, DSC, PLM, FT-IR	[73]
Lapatinib ditosylate	Soluplus^®^	Hot-melt extrusion and solvent evaporation	DSC, XRPD, SEM	[74]
n.a. (new chemical entity)	PVP	Solvent evaporation	TSDC, DSC	[46]
Naproxen	PVP	Solvent evaporation (spray drying)	M-DSC, FT-IR, XRPD	[75]
Nifedipine	PVP, HPMC, PHPA	Solvent evaporation (spray drying)	NMR, DSC	[48]
Posaconazole	Soluplus^®^, with the addition of PEG 4000, Poloxamer 188, Poloxamer 407 or TPGS	Hot-melt extrusion	DSC, M-DSC, SEM, AFM	[41]
Telaprevir	HPMC, HPMCAS, PVPVA	Solvent evaporation	AFM, AFM-IR, nanoTA, Fluorescence microscopy	[53]

AFM—Atomic Force Microscopy; AFM-IR—Nanoscale Infrared Spectroscopy; DSC—Differential Scanning Calorimetry; FT-IR—Fourier Transform Infrared Spectroscopy; FT-Raman—Fourier Transform Raman Spectroscopy; HEC—Hydroxyethyl Cellulose; HPC—Hydroxypropyl Cellulose; HPMC—Hydroxypropylmethyl Cellulose; HPMCAS—Hydroxypropylmethyl Cellulose Acetate Succinate; HPMCP—Hydroxypropylmethyl Cellulose Phthalate; HSM—Hot Stage Microscopy; IR imaging—Infrared imaging; M-DSC—Modulated-temperature Differential Scanning Calorimetry; μ-CT—Micro-computed Tomography; Na CMC—Sodium Carboxymethylcellulose; nanoTA—Nanoscale Thermal Analysis; NMR—Nuclear Magnetic Resonance; PAA—Polyacrylic Acid; PEG—Polyethylene Glycol; PHPA—α,β-poly(N-5-hydroxypentyl)-l-aspartamide; PLM—Polarized Light Microscopy; PVA—Poly(vinyl alcohol); PVP—Polyvinylpyrrolidone; PVPVA—Polyvinylpyrrolidone Vinyl Acetate; SEM—Scanning Electron Microscopy; TASC—Thermal Analysis by Structural Characterization; TPGS—d-α-Tocopheryl Polyethylene Glycol 1000 Succinate; XRPD—X-ray Powder Diffraction.

**Table 2:** The equations used for the estimation of the solubility parameter and its components in Hoy’s (1985) group contribution system [79].

Equations Used in the Calculation	Low Molecular Weight Substances	Amorphous Polymers
Additive molar functions	$Ft=\sum N_{i}F_{t,i}$	
	$Fp=\sum N_{i}F_{p,i}$	
	$V=\sum N_{i}V_{i}$	
	$\Delta_{T}=\sum N_{i}\Delta_{T,i}$	$\Delta_{T}{}^{\left(P\right)}={\sum N_{i}\Delta_{T,i}}^{\left(P\right)}$
Auxiliary equations	$Log\;\alpha =3.39\log(T_{b}/T_{cr})-0.1585-\log V$	$\alpha (P)=777\Delta_{T}{}^{\left(P\right)}/V$
	$T_{b}/T_{cr}=0.567+\Delta T-{\left(\Delta T\right)}^{2}$	$n=0.5/\Delta_{T}{}^{\left(P\right)}$
Calculation of total and partial solubility parameters	$\delta_{t}=(F_{i}+B)/V$	$\delta_{t}=(F_{i}+B/n)/V$
	$\delta_{p}=\delta_{t}{\left(\frac{1}{\alpha }\frac{F_{p}}{F_{t}+B}\right)}^{1/2}$	$\delta_{p}=\delta_{t}{\left(\frac{1}{\alpha^{\left(P\right)}}\frac{F_{p}}{F_{t}+B/n}\right)}^{1/2}$
	$\delta_{h}=\delta_{t}{\left[(\alpha -1)/\alpha \right]}^{1/2}$	$\delta_{h}=\delta_{t}{\left[(\alpha^{\left(P\right)}-1)/\alpha^{\left(P\right)}\right]}^{1/2}$
	$\delta_{d}={\left(\delta_{t}{}^{2}-\delta_{p}{}^{2}-\delta_{h}{}^{2}\right)}^{1/2}$	

*F_t_*—total molar attraction constant for each group; *F_p_*—polar molar attraction constant; *F_i_*—sum of molar attraction constants of constituent groups; *V*—molar volume of the molecule or repeated unit in the polymer; *Δ_T_*—Lydersen correction for non-ideality (values for low molecular substances have been provided by Lydersen [96], while values for polymers Δ_T_^(P)^ have been derived by Hoy; *T_b_*—boiling point; *T_cr_*—critical temperature; *B*—base value (*B* = 277).

## Abbreviations

AFM	Atomic Force Microscopy;
AFM-IR (nano IR)	Nanoscale Infrared Spectroscopy;
BCS	Biopharmaceutics Classification System
COMPASS	Condensed-phase Optimized Molecular Potentials for Atomistic Simulation Studies
DSC	Differential Scanning Calorimetry;
HEC	Hydroxyethyl Cellulose;
HPC	Hydroxypropyl Cellulose;
HPMC	Hydroxypropylmethyl Cellulose;
HPMCAS	Hydroxypropylmethyl Cellulose Acetate Succinate;
HPMCP	Hydroxypropylmethyl Cellulose Phthalate;
HSM	Hot Stage Microscopy;
MD	Molecular Dynamics;
M-DSC	Modulated-temperature Differential Scanning Calorimetry;
μ-CT	Micro-computed Tomography;
Na CMC	Sodium Carboxymethyl Cellulose;
nanoTA	Nanoscale Thermal Analysis;
NMR	Nuclear Magnetic Resonance;
PAA	Polyacrylic Acid;
PCFF	Polymer Consistent Force Field
PCRM	Pure Curve Resolution Method;
PC-SAFT	Perturbed-Chain Statistical Associating Fluid Theory;
PDF	Pair Distribution Functions;
PEG	Polyethylene Glycol;
PHPA	α,β-poly(N-5-hydroxypentyl)-l-aspartamide;
PLM	Polarized Light Microscopy;
PVA	Poly(vinyl alcohol);
PVP	Polyvinylpyrrolidone;
PVPVA	Polyvinylpyrrolidone Vinyl Acetate;
SEM	Scanning Electron Microscopy;
SSNMR	Solid State Nuclear Magnetic Resonance;
TASC	Thermal Analysis by Structural Characterization;
TEM	Transmission Electron Microscopy;
Tg	Glass Transition Temperature;
TPGS	d-α-Tocopheryl Polyethylene Glycol 1000 Succinate;
TSDC	Thermally stimulated depolarization current
